# NCOA4-Mediated Ferritinophagy: A Vicious Culprit in COVID-19 Pathogenesis?

**DOI:** 10.3389/fmolb.2021.761793

**Published:** 2021-12-15

**Authors:** Fengju Jia, Hongxia Liu, Shan Kang

**Affiliations:** ^1^ School of Nursing, Qingdao University, Qingdao, China; ^2^ Yantai Ludong Hospital (Shandong Provincial Hospital Group), Yantai, China; ^3^ Department of Laboratory, Qingdao Eighth People’s Hospital, Qingdao, China

**Keywords:** ferritinophagy, NCOA4, hyperferritinemia, iron, COVID-19

## Abstract

Coronavirus disease 2019 (COVID-19) is a global pandemic that has caused widespread loss of life. Notably, in this disease, severe inflammatory reactions characterized by cytokine storms are caused by severe acute respiratory syndrome coronavirus 2. The cytokine storms may promote hyper-ferritinemia which can further intensify the inflammation. Moreover, elevated ferritin levels trigger nuclear receptor coactivator 4 (NCOA4)-mediated ferritinophagy, in which ferritin is degraded and iron is released. Excess iron released from ferritinophagy can promote ferroptosis and cellular damage. Therefore, we propose that NCOA4-mediated ferritinophagy can be targeted to limit the ferroptosis and prevent the multi-organ damage and severity in COVID-19 patients.

## Introduction

Coronavirus disease 2019 (COVID-19), triggered by the novel severe acute respiratory syndrome coronavirus 2 (SARS-CoV-2), is a serious health concern and can be profoundly detrimental. In March 2020, the World Health Organization declared it as a pandemic. Although various vaccines have been developed, only limited number of therapeutic drugs is available. A significant proportion of patients with COVID-19 experience severe interstitial pneumonia, possibly resulting in acute respiratory distress syndrome and systemic inflammatory response syndrome.

### COVID-19 Systemic Inflammatory Response

COVID-19 systemic inflammatory reaction can be life-threatening due to its hyper-inflammation sustained by a cytokine storm. The cytokine storm is not only one of the earliest and most debilitating symptoms in patients with COVID-19 but also contributes to the disease severity at the later stage (Siddiqi and Mehra, 2020). Once inside the cells, the SARS-CoV-2 RNA is recognized by the pathogen recognition receptors, which trigger a downstream cascade of molecules leading to the activation of transcription factors, such as nuclear factor kappa B and interferon regulatory factor 3 (IRF-3) and the subsequent production of type 1 interferons and several other pro-inflammatory cytokines (e.g., interleukin [IL]-1β and IL-6) (Kawai and Akira, 2010; Conti et al., 2020; Yang et al., 2020). Considerably, elevated plasma levels of plasma pro-inflammatory cytokines, such as IL-6, IL-2, IL-7, IL-10, IFN-γ–induced protein 10 (IP-10), monocyte chemoattractant protein-1 (MCP-1), and macrophage inflammatory protein-1 alpha (MIP-1α), have been observed in some patients (Han et al., 2020; Karki et al., 2021). This expansion of an uncontrolled inflammatory response due to SARS-CoV-2 infection potentially leads to cell death by apoptosis, necrosis, or ferroptosis (a newly discovered type of programmed cell death), resulting in multi-organ damage in patients with COVID-19 (Banchini et al., 2021). Considering most of the acute and chronic deleterious effects of the SARS-CoV-2 infection are the consequences of hyper-inflammatory response of the virus, several inflammatory drugs have been used for the treatment of severe COVID-19 (Zhang et al., 2020).

## Hyper-Ferritinemia in COVID-19: A Part of Hyper-Ferritinemic Syndrome Spectrum

COVID-19–associated systemic inflammation is thought to be the part of the hyper-ferritinemic syndromes. COVID-19 shares many clinical and laboratory features such as lymphopenia, reduced NK cell numbers and activity, abnormal liver functions, and coagulopathy, with the other hyper-ferritinemic syndromes such as macrophage activation syndrome (MAS) and adult-onset Still’s disease (AOSD) (Rosario et al., 2013; Shoenfeld, 2020). Elevated pro-inflammatory cytokine and ferritin levels are the other hallmarks of hyper-ferritinemic syndromes common to COVID-19 (Colafrancesco et al., 2020).

Ferritin levels are elevated in various inflammatory disorders and directly correlated with poor prognosis and severity of the COVID-19 patients (Mehta et al., 2020). Although the source of circulating serum ferritin in COVID-19 has not yet been determined, macrophages contribute considerably to release ferritin *via* a non-classical secretory pathway (Cohen et al., 2010). A recent *in vitro* study showed that hepatocytes could actively secrete ferritin (Ghosh et al., 2004). Besides its active secretion in hepatocytes, serum ferritin is also derived by hepatic cell death (Colafrancesco et al., 2020). Furthermore, a key iron-regulatory hormone hepcidin could increase intracellular ferritin levels by sequestering iron in enterocytes and macrophages (Daher et al., 2017). Activated innate immunity and cytokine cascades can accelerate the expression of hepcidin (Drakesmith and Prentice, 2012; Cassat and Skaar, 2013). Interestingly, SARS-CoV-2 can mimic hepcidin by inducing ferroportin blockage and can result in the elevated ferritin levels independent of the inflammatory response (Cavezzi et al., 2020). Overall, patients with COVID-19 experience hyper-ferritinemia through the above mechanism.

## Hyper-Ferritinemia Amplifies Inflammation and the Cytokine Storm

The magnitude of the cytokine storm has a positive correlation with the serum ferritin levels. The high circulating ferritin levels in COVID-19 may not only reflect an acute phase response but also display other immunomodulatory functions (Recalcati et al., 2008; Edeas et al., 2020). Ferritin contains two subunits: L and H. H ferritin induces the expression of inflammatory cytokines such as IL-beta and IL-6 and promotes the myeloid and lymphocyte proliferation by stimulating a specific ferritin receptor TIM-2 (Ruscitti et al., 2016). Also, there is compelling evidence that the cytokine storm in hepatic cells is directly correlated with the extracellular ferritin levels (Ruddell et al., 2009). Thus, the mutual promotion of ferritin and inflammatory cytokine levels generates a vicious loop that constantly heightens the inflammatory state.

## Iron Dyshomeostasis in COVID-19

Ferritin is composed of 24 subunits, which can bind up to 4,500 atoms of iron. This makes ferritin one of the major iron storage proteins in the cells. It incorporates Fe^2+^ *via* ferritin iron pores and further oxidizes Fe^2+^ to Fe^3+^ by an H subunit inside the ferritin cage, leading to inert deposits of Fe^3+^ that are unavailable for intracellular use or generation of reactive oxygen species (ROS) (Philpott, 2018). Ferritin can also release Fe^3+^ and reduce it to Fe^2+^(Kidane et al., 2006), indicating that iron is critical in COVID-19 pathogenesis (Bellmann-Weiler et al., 2020; Edeas et al., 2020). Notably, iron overload directly influences several critical manifestations of COVID-19 such as systemic inflammation, hyper-ferritinemia, and immune dysfunction.

COVID-19–associated iron overload can be triggered by the release of the free iron from the damaged hemoglobin and ferritin catabolism. The process of viral infection is facilitated by several SARS-CoV-2 accessory viral proteins, including open reading frames (ORFs)-3, 10, and 8. The virus can interact with hemoglobin through angiotensin-converting enzyme 2 (ACE-2), CD-147, CD-26, and other receptors. The heme on the 1-beta chain of hemoglobin is attacked by ORF-8 and surface glycoprotein binding to porphyrin. Consequently, hemolysis and dysfunctional hemoglobin are engendered by SARS-CoV-2 infection, with or without forming a complex with the released heme (Cavezzi et al., 2020). Damage to hemoglobin causes dissociation of the porphyrins from iron and releases iron into the circulation resulting in the condition of iron overload (Cavezzi et al., 2020).

A retrospective cohort study showed that ferritin could lose a part of its inner iron content, giving rise to extremely high serum levels of free iron (Pretorius and Kell, 2014). Iron deposits derived from the ferritin lead to a poorly labile iron pool, indicating that the abundance of ferritin is a key factor governing iron homeostasis. Conversely, depletion of ferritin results in the release of iron into the labile iron pool, resulting in increased sensitivity to ferroptosis (Stockwell et al., 2020). Several studies have suggested that the selective autophagic turnover of ferritin (ferritinophagy) contributes to ferroptosis in fibroblasts and cancer cells (Hou et al., 2016). Although the role of ferritinophagy in COVID-19 remains unclear, its contribution in the pathological processes of neurodegeneration, cancer, ischemia/reperfusion injury, and urinary tract infections is well established (Tang et al., 2018).

Iron metabolism is objectively related to clinical syndromes during COVID-19 by inducing a series of biological events (Cavezzi et al., 2020). By interacting with molecular oxygen, excess intracellular iron can generate ROS (through Haber–Weiss and Fenton reactions), reactive nitrogen species (RNS), and reactive sulfur species (RSS) (Oexle et al., 1999). Through redox damage, mitochondrial dysfunction is favored, resulting in ferroptosis, multiple tissue damage, and subsequent fibrosis (Le Lan et al., 2005). During severe inflammatory conditions, excess iron can deteriorate the inflammatory reaction by inducing a severe pro-coagulant state (Pretorius and Kell, 2014). Moreover, higher iron levels may facilitate virus multiplication in the host cells (Drakesmith and Prentice, 2008). Iron depletion or chelation has been considered as a potential antiviral therapy to protect against excessive inflammatory responses and tissue damage by sequestering iron and preventing oxygen radical formation and lipid peroxidation in patients with COVID-19 (Perricone et al., 2020). In addition, the binding of SARS-CoV-2 to its receptors for entry into host cells can be prevented by lactoferrin, an iron chelator (Rainey et al., 2019; Chang et al., 2020).

## Nuclear Receptor Coactivator 4 (NCOA4)-Mediated Ferritinophagy in COVID-19

Recently, a ferroptosis signature has been observed in patients with COVID-19 (Jacobs et al., 2020). Ferroptosis inhibitors, such as ferrostatin-1, have been applied as potential drug candidates for COVID-19. Notably, NCOA4 has been reported to be the cargo receptor of ferritin in ferritinophagy (Dowdle et al., 2014; Mancias et al., 2014). NCOA4 binds to ferritin and delivers it to autophagosomes for ferritin degradation and iron release. ROS, RNS, RSS, and ferroptosis are generated by excess iron, consequently resulting in multiple tissue injury (Supplementary Figure S1). Additionally, Joseph et al. demonstrated that a direct association between a key surface arginine in ferritin and a C-terminal element in NCOA4 is required for the delivery of ferritin to the lysosome *via* autophagosomes (Mancias et al., 2015). Notably, NCOA4 depletion inhibits the delivery of ferritin to the lysosome, resulting in the disruption of ferritin degradation. The degradation of NCOA4 by HERC2, an E3 ubiquitin ligase, leads to suppressed ferritinophagy and elevated levels of ferritin (Mancias et al., 2015).

**FIGURE 1:**
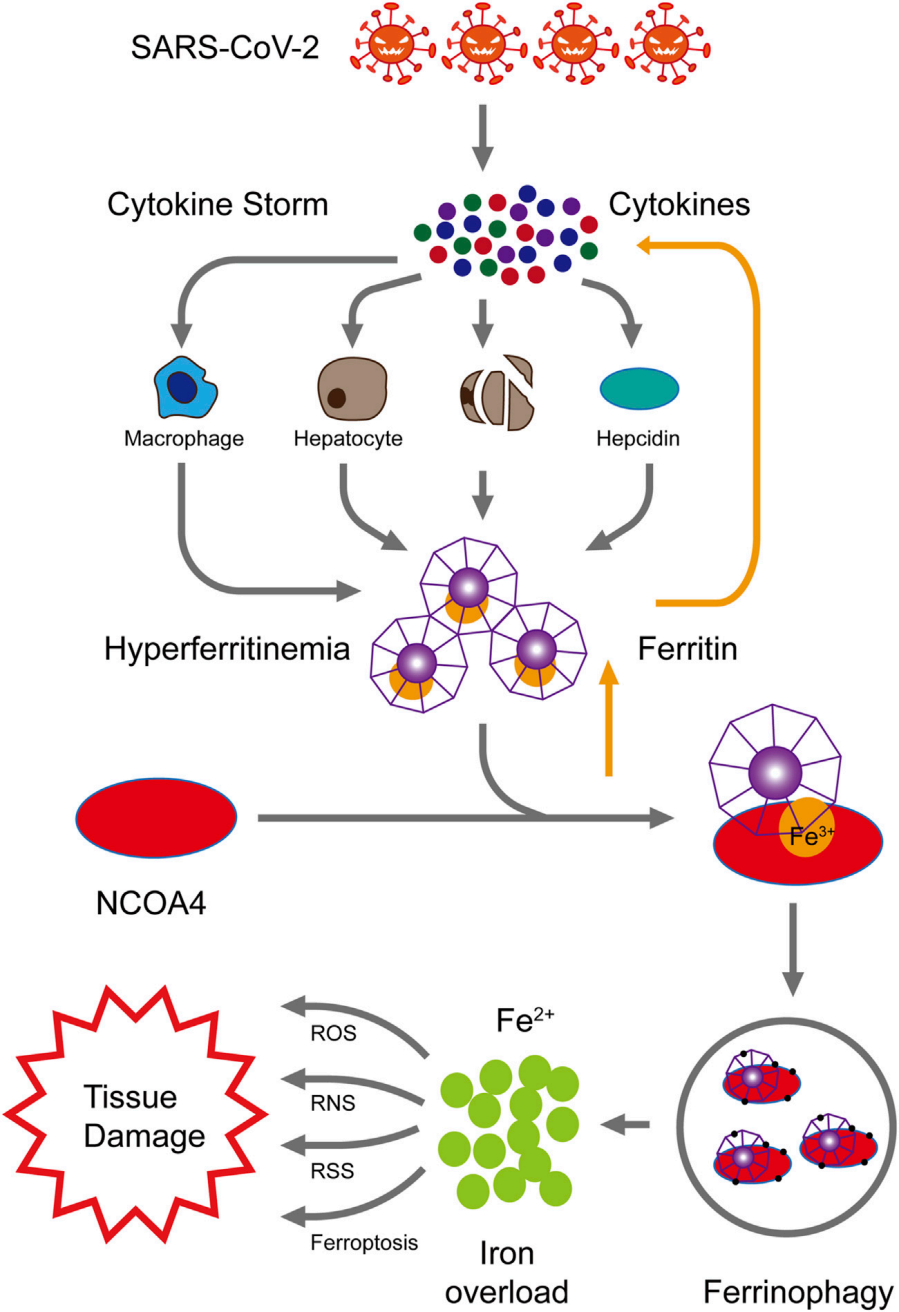
Proposed mechanism of NCOA4-mediated ferritinophagy in SARS-CoV-2 infection. High levels of inflammation characterized by cytokine storms are caused by SARS-CoV-2 infection. These cytokine storms cause hyper-ferritinemia, which further amplifies inflammation. The nuclear receptor coactivator 4 (NCOA4) binds to ferritin and delivers it to autophagosomes for ferritin degradation and iron release. Reactive oxygen species (ROS), reactive nitrogen species (RNS), reactive sulfur species (RSS), and ferroptosis are generated by the excess of intracellular iron, consequently resulting in multiple tissue injury.

Therefore, we hypothesize that cytokine storms caused by SARS-COV-2 infection may promote hyper-ferritinemia which can further intensify the inflammation. Elevated ferritin levels can trigger NCOA4-mediated ferritinophagy and may lead to ferroptosis, cell death, and organ damage. NCOA4-mediated ferritinophagy can be targeted to limit the ferroptosis and, therefore, prevent the multi-organ damage and severity in COVID-19 patients (Figure 1). Further studies should be conducted to confirm the involvement of NCOA4 and ferritinophagy in SARS-CoV-2 infection.
