# Supplementary figures and images for: NCOA4-Mediated Ferritinophagy: A Vicious Culprit in COVID-19 Pathogenesis?
Source: Front Mol Biosci. 2021 Dec 15;8:761793. doi: 10.3389/fmolb.2021.761793 (PMC8714652; doi:10.3389/fmolb.2021.761793)

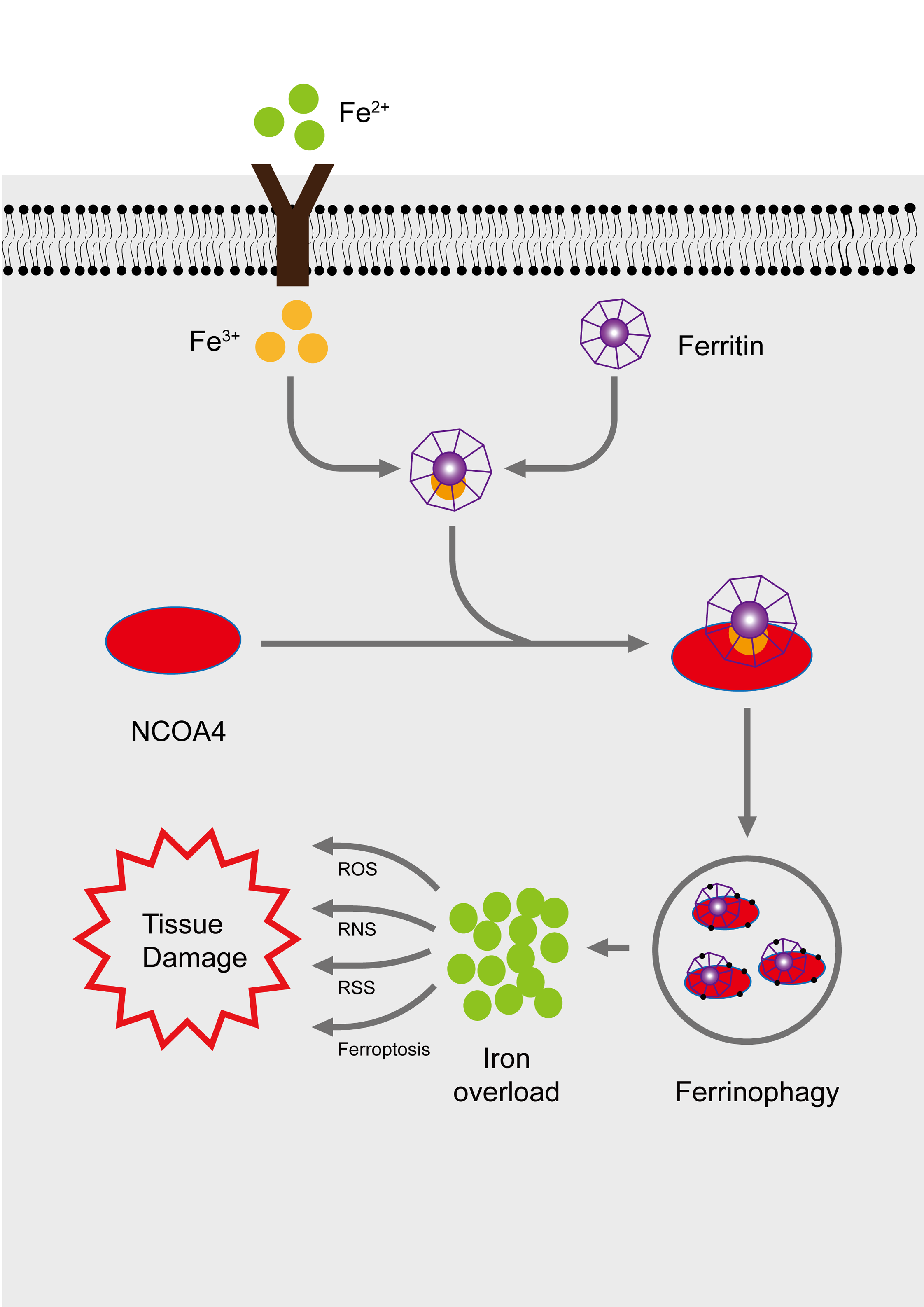

Supplement: Supplementary file 1 [file Image1.TIF]
